# Polyfunctional Ligand of the Clinical Setting: Thermodynamic Studies of the Equilibria of Tranexamic Acid in Aqueous Solutions

**DOI:** 10.1021/acsomega.6c02224

**Published:** 2026-05-26

**Authors:** Clemente Bretti, Rosalia Maria Cigala, Concetta De Stefano, Francesco Crea

**Affiliations:** Dipartimento di Scienze Chimiche, Biologiche, Farmaceutiche ed Ambientali, 18980 Università degli Studi di Messina, Viale F. Stagno d’Alcontres, 31, 98166 Messina, Italy

## Abstract

Tranexamic acid (*TXA*) is the main component of antihemorrhagic drugs but is also used as an ingredient in cosmetic formulations for its depigmenting action. The acid–base properties were investigated in aqueous solutions containing NaCl, KCl, and (CH_3_)_4_NCl, at different ionic strengths, at *T* = 298.15 ± 0.15 K and only in NaCl_(aq)_ at 283.15 ≤ *T*/K ≤ 318.15. The different behavior of *TXA* in the diverse media was explained by the formation of weak complexes with the components of the ionic media. The complexing ability of *TXA* toward Ca^2+^, Mg^2+^, Zn^2+^, and Sn^2+^, at *T* = 298.15 ± 0.15 K, was studied in NaCl_(aq)_ in the range of 0.15 ≤ *I*/mol dm^–3^ ≤ 1.0; the modeling of the formation constants with ionic strength was performed by an extended Debye–Hückel type equation and the Specific ion Interaction Theory. For all M^2+^/*TXA* systems, the common M­(*TXA*) species was obtained, and in the same experimental conditions, the stability trend observed is log*K*_Sn*TXA*_ > log*K*_Zn*TXA*_ > log*K*_Mg*TXA*_ > log*K*_Ca*TXA*_. The sequestering ability of *TXA* toward the metals was estimated by means of pL_0.5_. To complete the picture of the chemical behavior of *TXA*, some simulations in blood plasma were also performed.

## Graphical abstract

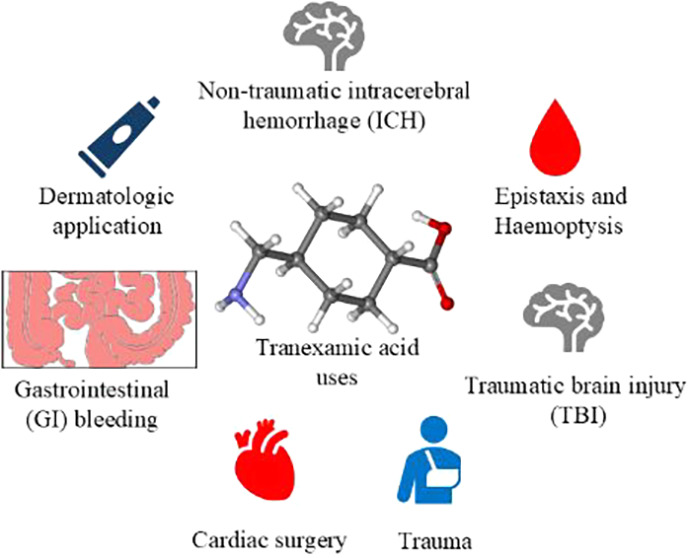

## 1 Introduction

Tranexamic acid (*TXA*) is a trans-stereoisomer of 4-(aminomethyl)­cyclohexanecarboxylic acid, a synthetic analog of lysine amino acid (see the structure in Figure 1). It has a wide range of applications, from cosmetology to the clinical fields, and plays an important role in the prevention and treatment of hemorrhagic conditions.

**Figure 1:**
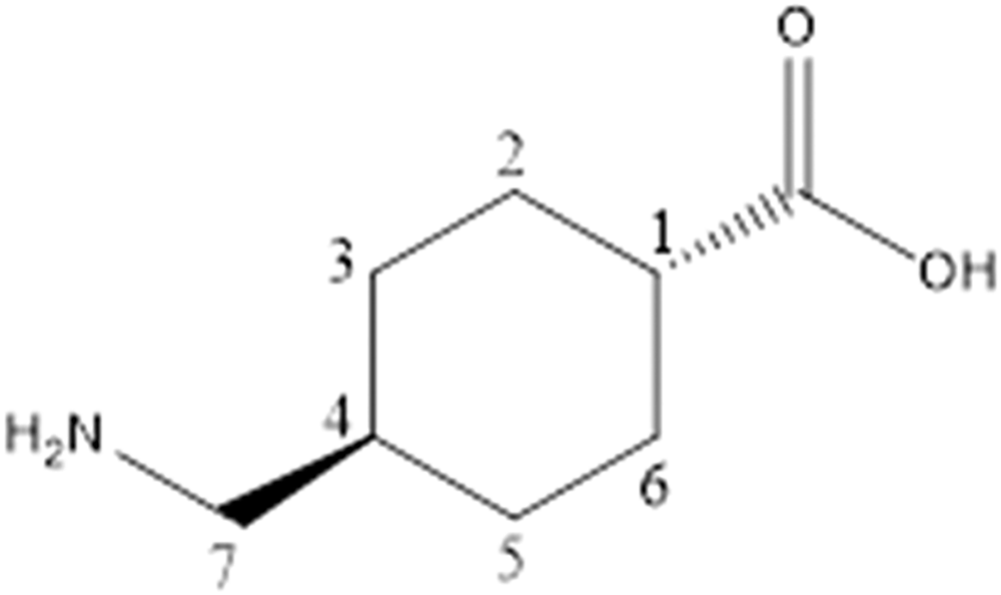
Tranexamic acid (*TXA*).

Different studies reported in literature have evidenced the efficiency of *TXA*; for example, Dunn et al.^1^ affirmed that “*TXA drug reduces postoperative blood losses and transfusion requirements in a number of types of surgery, with potential cost and tolerability advantages over aprotinin, and appears to reduce rates of mortality and urgent surgery in patients with upper gastrointestinal hemorrhage*”. Levy^2^ described the mechanism of action of tranexamic acid, and Reed and Woolley^3^ illustrated an overview of the applications of *TXA* in a wide clinical setting.

Goderecci et al.^4^ studied the effect of the topical application of tranexamic acid on “minimally” invasive joint surgical procedures, in which the articular cartilage is preserved. They concluded that when considering variables such as the type of treatment, exposure time, and concentration, a significant correlation exists between these factors and cell viability. No significant effect on cell viability was observed when the *TXA* exposure was limited to 10 min, whereas longer exposure times (24 and 48 h) resulted in a remarkable reduction in cell viability. Moreover, prolonged exposure to *TXA* may cause cartilage damage, and its use should be limited to a few minutes and at concentrations of 70 mg/mL or lower.

Tranexamic acid can be administered topically, orally, or as an injectable solution, depending on the treatment. Its biological activity may depend on the form in which it is administered; for this reason, the acid–base behavior and the complexing ability toward different metal cations (Ca^2+^, Mg^2+^, Zn^2+^ and Sn^2+^) were investigated under experimental conditions similar to physiological ones. The experimental investigations were performed in aqueous NaCl solutions, the main inorganic salt in biological and natural fluids, over a wide ionic strength range (0.10 ≤ *I*/mol dm^–3^ ≤ 1.0). To broaden the understanding of the thermodynamic behavior of *TXA* in aqueous solutions, measurements were also performed over the temperature range of 283.15 ≤ *T*/K ≤ 318.15. The study was extended to aqueous solutions containing KCl_(aq)_ and (CH_3_)_4_NCl_(aq)_ (0.15 ≤ *I*/mol dm^–3^ ≤ 1.0 and 0.15 ≤ *I*/mol dm^–3^ ≤ 2.5, respectively, at *T* = 298.15 ± 0.15 K), two additional supporting electrolytes with different interaction abilities toward *TXA*. This approach also allowed the determination of the stability constants of weak complexes formed between *TXA* and Na^+^, K^+^, and (CH_3_)_4_N^+^. The sequestering ability of tranexamic acid toward Ca^2+^, Mg^2+^, Zn^2+^, and Sn^2+^ was evaluated using the pL_0.5_ parameter under different ionic strength and pH conditions.

## 2 Experimental Section

### 2.1 Chemicals

Unless otherwise specified, all reagents used in the experiments were purchased from Fluka (Darmstadt, Germany) and have a purity of ≥95%.

Standard solutions of Ca^2+^, Mg^2+^, Zn^2+^, and Sn^2+^ were prepared by weighing the corresponding chloride salts (CaCl_2_·6H_2_O, MgCl_2_·6H_2_O, ZnCl_2_, and SnCl_2_·2H_2_O). Metal concentrations were determined by complexometric titrations using a standard EDTA solution.^5^ To prevent the oxidation of Sn^2+^ to Sn^4+^ and its hydrolysis, stock solutions were acidified with standard HCl_(aq)_ to obtain pH ∼2.0; a small piece of metallic tin was added, and purified N_2(g)_ was bubbled through the solution to exclude any O_2(g)_ traces.

*TXA* solutions were prepared from the commercial product without further purification, and the purity was verified by alkalimetric titrations, resulting always at >99.5%. Hydrochloric acid, as well as sodium, potassium, and tetramethylammonium hydroxide solutions, was standardized using sodium carbonate and potassium hydrogen phthalate, both previously dried at *T* = 383.15 K. The NaOH, KOH, and (CH_3_)_4_NOH solutions were preserved from CO_2_ through soda lime traps.

Aqueous solutions of NaCl, KCl, and NaNO_3_ (for voltammetry), used as background electrolytes, were prepared by weighing the pure salt previously dried in an oven at *T* = 383.15 K for 2 h. Aqueous (CH_3_)_4_NCl solutions were prepared by weighing the salt after recrystallization from methanol and drying under vacuum.^6^ For voltammetric measurements, Sn^2+^ solutions were prepared by dilution of a 1000 ppm standard Sn^2+^ solution in HNO_3_, purchased from Sigma-Aldrich. Details such as the CAS number, purification process, and mass concentration are listed in Table 1.

**Table 1:** Sample Description

chemical	CAS no.	purification	mass % concentration
calcium chloride hexahydrate	7774–34–7	no	≥95%^a^
magnesium chloride hexahydrate	7791–18–6	no	≥99%^a^
zinc chloride	7646–85–7	no	≥98%^a^
tin(II) chloride dihydrate	10025–69–1	no	≥98%^a^
hydrochloric acid	7647–01–0	no	∼37%^b^
sodium hydroxide	1310–73–2	no	≥97%^b^
potassium hydroxide	1310–58–3	no	≥97%^b^
sodium carbonate	497–19–8	no	≥99.5%
potassium phthalate monobasic	877–24–7	no	≥99.5%
sodium chloride	7647–14–5	no	≥99%
sodium nitrate	7631–99–4	no	≥99.0%
potassium chloride	7447–40–7	no	≥99%
tetramethylammonium chloride	75–57–0	yes	purified after recrystallization^6^ ^,^ ^c^
tetramethylammonium hydroxide	75–59–2	no	25%^b^
tranexamic acid	1197–18–8	no	>99.5%

a The metal concentration was determined by complexometric titrations with EDTA.^5^

b The concentration was determined by alkalimetric and acidimetric titrations.

c The concentration, after recrystallization, was determined by HPLC and resulted to be ≥99%.

All solutions were prepared using analytical-grade water (*R* = 18 MΩ cm^–1^) and class A glassware.

### 2.2 Potentiometric Measurements: Apparatus and Procedure

Potentiometric measurements were performed using an automatic titration system (Metrohm Titrando) equipped with a Dosino dispenser and a combined glass electrode. The instrument was connected to a PC and administered by Metrohm TiAmo 2.2 software, which managed the titrant delivery, data acquisition, and electromotive force (e.m.f.) stability. The accuracy of the system was ±0.15 mV for e.m.f. and ±0.002 cm^3^ for titrant volume.

For the study of acid–base properties, a volume of 25 cm^3^ of a solution containing *TXA*, inorganic acid (HCl), and the supporting electrolyte (NaCl, KCl, or (CH_3_)_4_NCl) at the desired ionic strength was titrated with standard hydroxide solutions (NaOH, KOH, or (CH_3_)_4_NOH). For complexation studies, the solutions also contained the metal ions. Potentiometric measurements (both protonation and complexation) were carried out in thermostated glass-jacketed cells at controlled temperatures: *T* = 298.15 ± 0.15 K for the metal interaction and protonation studies in KCl and (CH_3_)_4_NCl media and 283.15 ≤ *T*/K ≤ 318.15 in NaCl solutions (for protonation only). All experiments were conducted under magnetic stirring, with continuous bubbling of purified N_2_ through the solutions to avoid O_2_ and CO_2_ inside. Titrations were performed until the formation of a precipitate, depending on component concentrations, ionic strength, temperature, and the acid–base properties of the metal ions.

For each experiment, independent titrations of HCl with standard hydroxide solutions were carried out at the same experimental conditions (i.e., ionic strength, ionic medium, and temperature) of the test solutions, in order to determine the standard electrode potential (*E* ^0^) and the ionic product of the water (p*K*_w_). The experimental details are listed in Table 2.

**Table 2:** Experimental Conditions of the *TXA* Systems, at *p* = 0.1 MPa

system	*c*_M_ ^a^	*c_TXA_* ^b^	pH range	*I* ^c^	ionic medium	*T*/K^d^	technique^e^
H^+^/*TXA*		(5.0–15.0)·10^–3^	2.0–10.8	0.15–4.7	NaCl	283.15–318.15	Pot
		(1.0–5.0)·10^–3^	2.0–10.8	0.15–1.0	KCl	298.15	Pot
		(5.0–15.0)·10^–3^	2.0–10.8	0.10–2.5	(CH_3_)_4_NCl	298.15	Pot
Ca^2+^/*TXA*	(1.0–3.0)·10^–3^	(1.0–6.9)·10^–3^	2.0–10.5	0.15–1.0	NaCl	298.15	Pot
Mg^2+^/*TXA*	(1.0–2.0)·10^–3^	(1.0–8.0)·10^–3^	2.0–10.5	0.15–1.0	NaCl	298.15	Pot
Zn^2+^/*TXA*	(1.0–3.0)·10^–3^	(1.0–7.2)·10^–3^	2.0–8.0	0.15–1.0	NaCl	298.15	Pot
Sn^2+^/*TXA*	10^–7^–10^–5^	(3.0–5.0)·10^–3^	2.0–4.5	0.10–1.0	NaNO_3_	298.15	Volt

a *c*_M_ = metal concentration in mol dm^–3^.

b *c_TXA_* = tranexamic acid concentration in mol dm^–3^.

c *I* = ionic strength in mol dm^–3^.

d *T* = temperature in Kelvin.

e Pot = potentiometry and Volt = voltammetry. Standard uncertainties (*u*): *u*(*T*) = 0.1 K, *u*(*I*) = 0.001 mol dm^–3^, and *u*(*p*) = 1 kPa.

### 2.3 Voltammetric Measurements: Apparatus and Procedure

Differential pulse anodic stripping voltammetry (DP-ASV) was used to study the Sn^2+^/*TXA* system, at *T* = 298.15 ± 0.15 K, in NaNO_3(aq)_ solutions with ionic strengths in the range of 0.10 ≤ *I*/mol dm^–3^ ≤ 1.0 mol dm^–3^. The apparatus consists of a Metrohm 663 VA stand workstation, equipped by three electrodes: a multimode mercury electrode working in static mercury drop electrode mode (SMDE), a glassy carbon auxiliary electrode, and a double junction Ag/AgCl/KCl (3.0 mol dm^–3^) reference electrode. The workstation was connected to a μAutolab type III potentiostatic/galvanostatic (Eco Chemie) with an IME663 interface (Eco Chemie). The voltammetric system was controlled by GPES v. 4.9 software (Eco Chemie).

DP-ASV measurements were carried out on solutions (total volume: 25 cm^3^) containing known amounts of Sn^2+^, inorganic acid (to adjust the pH value to 2.0), and NaNO_3_ to achieve the preestablished ionic strength values. Different amounts of *TXA* were added to obtain the chosen total ligand concentration in the cell (further experimental details are provided in Table 2), varying the *c*_Sn_/*c*_*TXA*_ ratios. The free hydrogen ion concentration was measured using a Metrohm 750 combined glass electrode, previously calibrated as described in Section 2.2.

For each voltammetric titration, 30–40 voltammograms were recorded in the pH range of 2.0 ≤ pH ≤ 4.5 (by adding standard NaOH), with the following setup: (i) bubbling purified N_2_ through the solution for 300 s; (ii) applying a deposition potential of −1.8 V for 40 s under stirring; (iii) a rest time of 20 s; and (iv) recording the voltammogram in the range from −0.6 to −0.254 V with a scan rate of 7.5 mV s^–1^ and a step potential of 1.5 mV. The modulation amplitude was 25 mV, with a modulation time of 0.05 s and an interval of 0.5 s.

### 2.4 Equilibria and Modeling Studies

The acid–base properties of *TXA* are associated with two protonable sites: a primary amine group (−NH_2_) and a carboxylic one (−COOH). The protonation equilibria can be expressed by the following stepwise equations

1 $$H^{+}+\mathrm{TX}A^{-}=H\mathrm{TXA},,,,K_{1}^{H}$$

2 $$H^{+}+H\mathrm{TXA}=H_{2}\mathrm{TX}A^{+},,,,K_{2}^{H}$$

or by the overall general equilibrium

$$iH^{+}+\mathrm{TX}A^{-}=H_{i}\mathrm{TX}A^{\left(-1+i\right)},,(with\;i=1\;or\;2),,\beta_{i}^{H}$$ 3

For a correct speciation study of the complexing ability of *TXA* toward the metal cations, the acid–base properties of each metal must also be considered; these can be expressed by the general equation

4 $${pM}^{2+}+{rH}_{2}O=M_{p}{{\left(\mathrm{OH}\right)}_{r}}^{\left(2p-r\right)}+{rH}^{+},,,,{\beta_{r}}^{\mathrm{OH}}$$

For the Sn^2+^, the formation of the weak SnCl*_n_* complexes and the possible formation of the insoluble Sn­(OH)_2(s)_ species were also considered by means of the equilibria

5 $${\mathrm{Sn}}^{2+}+{nCl}^{-}={{\mathrm{SnCl}}_{n}}^{\left(2-n\right)},,,,\beta_{n}$$

and

6 $$\mathrm{Sn}{\left(\mathrm{OH}\right)}_{2\left(s\right)}+2H^{+}={\mathrm{Sn}}^{2+}+2H_{2}O,,,,K_{s}$$

Considering the equilibria described above, the formation (or stability) constants of the different M^2+^/*TXA* complex species can be expressed as follows

7 $$M^{2+}+\mathrm{TX}A^{-}=M\mathrm{TX}A^{\left(2-1\right)},,,,\beta$$

$$M^{2+}+\mathrm{TX}A^{-}+{rH}_{2}O=M\mathrm{TXA}{{\left(\mathrm{OH}\right)}_{r}}^{\left(2-1-r\right)}+{rH}^{+},,\beta_{11-r}$$ 8

Two different approaches were employed to model the variation of the formation constants with ionic strength, namely an extended Debye–Hückel type equation and the Specific ion Interaction Theory (SIT).

The Debye–Hückel type equation can be expressed in the general form ([Disp-formula eq9])­

9a $$\log\beta =\log\beta^{0}-z^{*}\cdot A\left(I^{1/2}/\left(1+1.5\cdot I^{1/2}\right)\right)+\left(C\right)\cdot I+\left(M\right)$$

where logβ ^0^ is the equilibrium (protonation or stability) constant at infinite dilution (i.e., extrapolated to zero ionic strength) and z* = ∑(charge)_reactants_^2^ – ∑(charge)_products_^2^.

*A* is the Debye–Hückel term, which at *T* = 298.15 K is equal to 0.51; when investigations are also carried out at different temperatures, its dependence on *T*/K can be expressed as

9b $$A=0.51+\left(0.856\cdot \left(T-298.15\right)+0.00385\cdot {\left(T-298.15\right)}^{2}\right)/1000)$$

where 0.856 and 0.00385 are empirical parameters. The parameter (*C*) accounts for the variation of the equilibrium constant (logβ) with *I*/mol dm^–3^.

The literature^7–10^ reports that when investigations are performed at high ionic strength (*I* > 1.0 mol dm^–3^), it is necessary to expand the Debye–Hückel type equation by including additional polynomial terms (i.e., (*D*)·*I* ^3/2^ and (*E*)·*I* ^2^). The *C* term in eq 9 (as well as *D* and *E* parameters) can be explicated as reported in the following eq 11 ^7–10^

10a $$C=c_{0}p^{*}+c_{1}z^{*}$$

where p* = ∑*p*_reactants_ – ∑*p*_products_ (*p* = stoichiometric coefficient of the components); the *c*_0_ and *c*_1_ values, in noninteracting media (for example, in (C_2_H_5_)_4_NI) and at *T* = 298.15 K, are 0.11 and 0.20, respectively.^11^ The *C*, *D*, and *E* parameters depend on the charges of the reactants and products and on the stoichiometry of the reaction and are independent of the chemical nature of the components.^7,8^ In previous studies, it was demonstrated that the *C* parameter can be sufficient to model the variation of the equilibrium constants up to high ionic strengths (*I* ∼ 6.0 mol dm^–3^) when expressed as in eq 12 ^11^

10b $$C=c_{\infty }+\left(c_{0}-c_{\infty }\right)/\left(I+1\right)$$

in which *c*_∞_ is the value of *C* at *I* → ∞ and *c*_0_ is the corresponding value at *I* → 0. If the equilibrium constants and ionic strength are expressed on the molal scale (mol kg^–1^(H_2_O)), eq 9 becomes equivalent to that used in the Specific ion Interaction Theory (SIT), and *C* ≃ Δε. Moreover, similar to eq 12, the Δε parameter can also be expressed as in eq 13

11 $$\Delta \varepsilon =\Delta \varepsilon_{\infty }+\left(\Delta \varepsilon_{0}-\Delta \varepsilon_{\infty }\right)/\left(I+1\right)$$

where Δε_0_ and Δε_∞_ have the same meaning as the parameters in eq 12 but are valid for the molal concentration scale.

The variation of the protonation constants with temperature was modeled using the Clarke and Glew eq 14 ^12^

$$\left(M\right)=\Delta H^{0}-z^{*}(1.5\cdot (I^{1/2}/\left(1+1.5\cdot I^{1/2}\right)+\Delta \varepsilon^{\prime }\cdot I+\Delta C_{p}\cdot \left(1/298.15-1/T\right)\cdot 52.23$$ 12

where Δ*H* ^0^/kJ mol^–1^ is the protonation enthalpy at infinite dilution on the molal concentration scale, Δε′ is the parameter for its dependence on ionic strength, while Δ*C*_p_ is expressed in J K^–1^ mol^–1^, and 52.33 is 1/(*R* ln 10). The three parameters in eq 14 can also be determined for the molar concentration scale, in which case they are substituted by the parameters *A*′, *B*′, and *C*′, which can be considered as gradient of temperature parameters.

Regarding the voltammetric measurements, the peak potential shift due to the formation of labile complex species at the *i*th pH value is given by

13 $${\Delta E}_{p}=E_{p}^{\mathrm{free}}-E_{p}^{\mathrm{comp}}=\frac{\mathrm{RT}}{\mathrm{nF}}\ln\left(\frac{c_{{\mathrm{Sn}}^{2+}}}{\left[{Sn}^{2+}\right]}\right)+\frac{\mathrm{RT}}{\mathrm{nF}}\ln\left(\frac{I_{p}^{\mathrm{comp}}}{I_{p}^{\mathrm{free}}}\right)$$

Δ*E*_p_ represents the peak shift due to the difference between the peak potential of the free and complexed metal ions, *E*_p_^free^ and *E*_p_^comp^, respectively. The *I*_p_^comp^ and *I*_p_^free^ are the current intensities, (peak heights) with and without the ligand in solution, respectively. The *c*_Sn_^2+^ and [Sn^2+^] are the analytical and the free metal ion concentrations, respectively, at the *i*th pH value.

The *E*_p_^free^, *E*_p_^comp^, *I*_p_^comp^, and *I*_p_^free^ in eq 15 are collected from the voltammetric scans, while the free metal ion concentration is determined by resolving the mass balance equations. It can be assumed that at high *c_TXA_*:*c*_Sn_^2+^ ratios and low metal concentrations, the formation of polynuclear species is avoided. Based on this assumption, the free [Sn^2+^] concentration at the *i*th pH is given by

$$\left[{\mathrm{Sn}}^{2+}\right]=\frac{c_{{\mathrm{Sn}}^{2+}}}{1+\sum \beta_{\mathrm{Sn}H_{i}{\mathrm{TXA}}_{m}}{\left[H\right]}^{i}{\left[\mathrm{TXA}\right]}^{m}}$$ 14

and

$$\left[{\mathrm{TXA}}^{-}\right]=\frac{c_{{\mathrm{TXA}}^{-}}}{1+\sum \beta {\left[H\right]}^{i}}$$ 15

where β is the overall protonation constant of the ligand.

The summation in eq 16 accounts for the formation of hydrolytic species when *i* < 0 and *m* = 0.

### 2.5 Computer Programs Used for Calculations

The parameters of the potentiometric titrations, namely the standard electrode potential (*E* ^0^), ionic product of water (p*K*_w_), and the analytical concentration of the ligand and of the inorganic acid, were calculated using the ESAB2M computer program.^13^ The experimental data obtained from the potentiometric titrations (electromotive force (mV)/volume (cm^3^)), which are useful for determining the protonation and complex formation constants, were processed using the BSTAC program.^14^ The distribution of the species was analyzed using the HySS program,^15^ which allowed for the calculation of the formation percentages of the species and to draw the distribution diagrams. The dependence of the protonation and complex formation constants on ionic strength, modeled using the extended Debye–Hückel type equation and the Specific ion Interaction Theory (SIT) (see Section 2.4), was carried out using the LIANA computer program.^16^ This last program was also employed for the elaboration of the voltammetric data.

The selection of the species comprising the speciation models of all M^2+^/*TXA* systems was carried out by applying some accuracy and reliability criteria, in particular: simplicity and probability of the species, percentages of formation in the measured pH range, statistical parameters (e.g., standard deviation of the constants and fit), and the ratios between the variances of the chosen model and the other checked ones.

## 3 Results and Discussion

### 3.1 Acid–Base Properties of Tranexamic Acid

The protonation constants of *TXA* were determined under different experimental conditions, namely, NaCl_(aq)_ at 0.15 ≤ *I*/mol dm^–3^ ≤ 4.6 and 283.15 ≤ *T*/K ≤ 318.15, KCl_(aq)_ at 0.15 ≤ *I*/mol dm^–3^ ≤ 1.0 and *T* = 298.15 K, and (CH_3_)_4_NCl_(aq)_ at 0.10 ≤ *I*/mol dm^–3^ ≤ 2.5 and *T* = 298.15 K. Using the BSTAC program, it was possible to determine the protonation constants of the two protonable groups, namely, the carboxylic and primary amine groups; the obtained results are reported for both the molar (mol dm^–3^) and molal (mol kg^–1^) concentration scales in Table 3.

**Table 3:** Experimental Protonation Constants^a^ of Tranexamic Acid, in NaCl_(aq)_, KCl_(aq)_, and (CH_3_)_4_NCl_(aq)_, at Different Ionic Strengths and Temperatures, at *p* = 0.1 MPa

*T*/K^b^	*I* ^c^/mol dm^–3^	log*K*_1_^H^ ^d^	log*K*_2_^H^ ^d^	*I*/mol kg^–1^	log*K*_1_^H^	log*K*_2_^H^
		molar scale		molal scale		
NaCl						
283.15	0.150	11.075 ± 0.014^e^	4.393 ± 0.006	0.151	11.074 ± 0.016	4.391 ± 0.005
	0.500	11.149 ± 0.013	4.412 ± 0.006	0.501	11.145 ± 0.015	4.408 ± 0.005
	0.966	11.273 ± 0.005	4.470 ± 0.004	0.982	11.265 ± 0.003	4.462 ± 0.003
	1.960	11.521 ± 0.003	4.639 ± 0.004	2.031	11.505 ± 0.002	4.623 ± 0.002
	2.905	11.744 ± 0.003	4.822 ± 0.003	3.070	11.720 ± 0.002	4.798 ± 0.002
	4.000	11.996 ± 0.005	5.046 ± 0.004	4.336	11.961 ± 0.004	5.011 ± 0.003
291.15	0.150	10.705 ± 0.012	4.377 ± 0.006	0.151	10.703 ± 0.013	4.375 ± 0.004
	0.500	10.779 ± 0.010	4.394 ± 0.006	0.501	10.774 ± 0.011	4.389 ± 0.004
	0.974	10.906 ± 0.002	4.449 ± 0.002	0.992	10.897 ± 0.002	4.440 ± 0.001
	1.988	11.162 ± 0.002	4.614 ± 0.002	2.067	11.145 ± 0.001	4.597 ± 0.001
	2.939	11.389 ± 0.002	4.792 ± 0.002	3.119	11.375 ± 0.001	4.766 ± 0.001
	3.914	11.616 ± 0.004	4.983 ± 0.003	4.250	11.580 ± 0.003	4.947 ± 0.001
298.15	0.150	10.413 ± 0.012	4.374 ± 0.003	0.151	10.411 ± 0.012	4.371 ± 0.003
	0.488	10.483 ± 0.002	4.388 ± 0.001	0.494	10.477 ± 0.001	4.382 ± 0.001
	0.965	10.612 ± 0.002	4.440 ± 0.001	0.985	10.603 ± 0.002	4.430 ± 0.001
	1.911	10.853 ± 0.002	4.586 ± 0.002	1.989	10.835 ± 0.001	4.568 ± 0.001
	2.752	11.057 ± 0.002	4.736 ± 0.003	2.918	11.032 ± 0.001	4.710 ± 0.001
	4.684	11.512 ± 0.004	5.105 ± 0.005	5.207	11.466 ± 0.003	5.059 ± 0.002
310.15	0.150	9.979 ± 0.012	4.396 ± 0.005	0.151	9.975 ± 0.013	4.392 ± 0.004
	0.500	10.053 ± 0.009	4.407 ± 0.004	0.501	10.046 ± 0.008	4.400 ± 0.004
	0.993	10.187 ± 0.004	4.456 ± 0.002	1.019	10.176 ± 0.003	4.444 ± 0.002
	1.997	10.446 ± 0.003	4.602 ± 0.002	2.094	10.425 ± 0.001	4.581 ± 0.001
	2.954	10.681 ± 0.002	4.764 ± 0.003	3.165	10.651 ± 0.001	4.733 ± 0.001
	3.915	10.912 ± 0.001	4.935 ± 0.004	4.295	10.872 ± 0.002	4.895 ± 0.002
318.15	0.150	9.738 ± 0.013	4.429 ± 0.006	0.151	9.732 ± 0.015	4.423 ± 0.006
	0.500	9.812 ± 0.010	4.437 ± 0.007	0.501	9.803 ± 0.011	4.428 ± 0.006
	0.991	9.946 ± 0.007	4.482 ± 0.004	1.021	9.933 ± 0.004	4.469 ± 0.004
	1.998	10.208 ± 0.005	4.621 ± 0.003	2.103	10.186 ± 0.002	4.598 ± 0.003
	2.889	10.430 ± 0.004	4.765 ± 0.004	3.104	10.399 ± 0.001	4.733 ± 0.002
	3.936	10.685 ± 0.004	4.944 ± 0.006	4.339	10.642 ± 0.002	4.902 ± 0.003
KCl						
298.15	0.149	10.380 ± 0.002	4.396 ± 0.002	0.150	10.377 ± 0.005	4.392 ± 0.003
	0.245	10.379 ± 0.002	4.406 ± 0.003	0.247	10.375 ± 0.006	4.401 ± 0.005
	0.484	10.421 ± 0.003	4.431 ± 0.005	0.492	10.414 ± 0.006	4.424 ± 0.011
	0.723	10.485 ± 0.003	4.457 ± 0.008	0.740	10.475 ± 0.008	4.446 ± 0.016
	0.965	10.562 ± 0.005	4.482 ± 0.010	0.995	10.549 ± 0.017	4.468 ± 0.021
(CH_3_)_4_NCl						
298.15	0.109	10.412 ± 0.001	4.370 ± 0.001	0.111	10.406 ± 0.001	4.363 ± 0.001
	0.109	10.412 ± 0.001	4.370 ± 0.001	0.111	10.406 ± 0.001	4.363 ± 0.001
	0.500	10.456 ± 0.002	4.385 ± 0.006	0.530	10.431 ± 0.020	4.360 ± 0.008
	0.878	10.508 ± 0.002	4.442 ± 0.001	0.973	10.463 ± 0.002	4.397 ± 0.002
	0.890	10.510 ± 0.002	4.444 ± 0.001	0.987	10.465 ± 0.002	4.399 ± 0.002
	1.722	10.587 ± 0.002	4.632 ± 0.002	2.119	10.497 ± 0.002	4.541 ± 0.003
	1.728	10.587 ± 0.002	4.633 ± 0.002	2.128	10.497 ± 0.002	4.542 ± 0.003
	2.526	10.635 ± 0.002	4.846 ± 0.003	3.472	10.497 ± 0.003	4.708 ± 0.006
	2.539	10.636 ± 0003	4.850 ± 0.003	3.497	10.497 ± 0.003	4.711 ± 0.006

a Refer to the equilibria reported in eqs 1 and 2.

b *T* = temperature in Kelvin.

c *I* = ionic strength.

d log*K*_1_^H^ and log*K*_2_^H^ are the protonation constants of the first and second protonation steps, respectively.

e ±Std. Dev. on a single constant value (obtained by a set of 3–5 measurements). Standard uncertainties (*u*): *u*(*T*) = 0.1 K, *u*(*I*) = 0.001 mol dm^–3^, and *u*(*p*) = 1 kPa.

The protonation constants show different trends depending on the supporting electrolyte; for example, the log*K*_1_^H^ value, regarding the protonation of the primary amine group, decreases from NaCl_(aq)_ to KCl_(aq)_ and further decreases in (CH_3_)_4_NCl_(aq)_. In the case of the protonation of the carboxylic group, the log*K*_2_^H^ value has an opposite trend with respect to log*K*_1_^H^; in fact, it increases from NaCl_(aq)_ to KCl_(aq)_, further increasing in (CH_3_)_4_NCl_(aq)_. This trend is shown in Figure 2, for *I* = 0.9 mol dm^–3^ and *T* = 298.15 ± 0.15 K.

**Figure 2:**
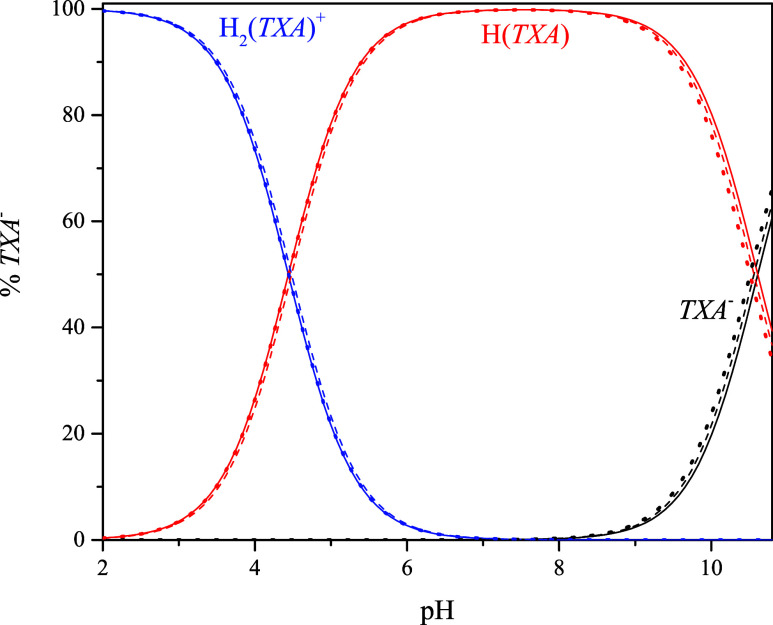
Distribution diagram of the protonated species of the *TXA* in the three different ionic media: NaCl (solid line), KCl (dash line), and (CH_3_)_4_NCl (dot line), *c*_*TXA*_ = 5.0 ·10^–3^ mol dm^–3^, at *I* = 0.9 mol dm^–3^ and *T* = 298.15 ± 0.15 K.

Regarding the effect of ionic strength on the protonation constants, it can be noted that, in general, the log*K* ^H^ *_i_* values increase with increasing the ionic strength value in all ionic media investigated. An exception is represented by KCl_(aq)_, in which log*K*_1_^H^ decreases with increasing ionic strength up to ${\mathrm{I̅}=0.48\;\mathrm{mol}\;\mathrm{dm}}^{-3}$ and subsequently increases by increasing the ionic strength. The trend of the protonation constants of *TXA* with respect to ionic strength in different ionic media and at *T* = 298.15 K, is shown in Figure 3.

**Figure 3:**
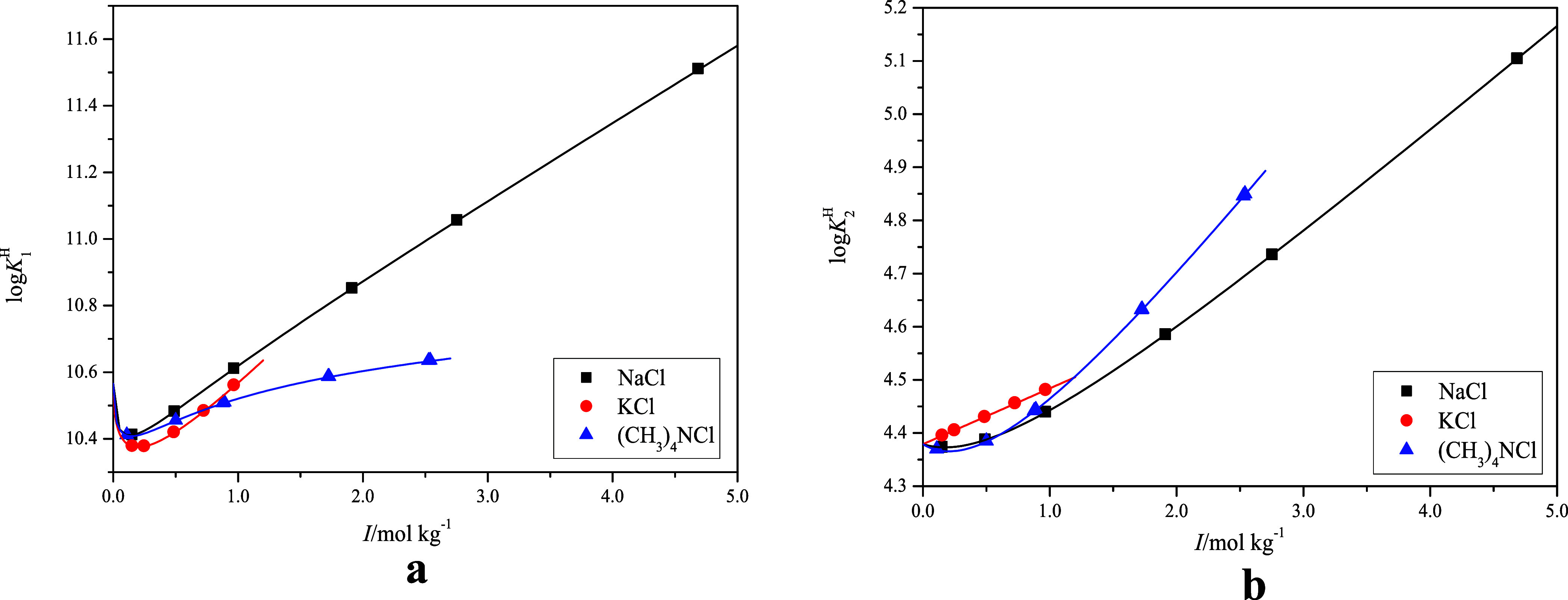
Trend of the first (a) and the second (b) protonation steps of the *TXA* with respect to the ionic strength in the different ionic media and at *T* = 298.15 K, following the ionic strength dependence parameters of eq 13, reported in Table 5.

A study on the acid–base properties of tranexamic acid in the literature^17^ reports the protonation constant values of *TXA* obtained at *T* = 298.1 K in NaNO_3(aq)_, as a supporting electrolyte, at *I* = 0.1 mol dm^–3^: log*K*_1_^H^ = 10.69 ± 0.04 and log*K*_2_^H^ = 4.76 (logβ_2_^H^ = 15.45 ± 0.07). Comparing these data with those obtained here in NaCl at the same ionic strength (*I* = 0.1 mol dm^–3^), log*K*_1_^H^ = 10.74 and log*K*_2_^H^ = 4.33, it is observed that the value of log*K*_1_^H^ (protonation constant of the amino group) is the same within the experimental error, while a difference of 0.43 unit is obtained for the log*K*_2_^H^ value associated with the protonation constant of the COOH group. This difference cannot be attributed to the different supporting electrolytes used (NaNO_3_ versus NaCl), since the potentially interacting cation is always Na^+^. The only difference is attributable to the anion (Cl^–^ versus NO_3_^–^).

It is well-known that Cl^–^ can form highly stable ion pairs with amino groups, while this is not observed with NO_3_^–^; the differences between the *TXA* protonation constant values in the two media, therefore, are not due to their different nature, especially the protonation constant of the carboxyl group. Another study,^18^ on the complexation of Al^3+^ by *TXA*, determined the protonation constants of the ligand by ^1^H NMR measurements, in NaCl_(aq)_ at *I* = 0.15 mol dm^–3^ and *T* = 298.15 K: log*K*_1_^H^ = 10.72 ± 0.02 and log*K*_2_^H^ = 4.43 (logβ_2_^H^ = 15.15 ± 0.04). As it can be seen, the ^1^H NMR data are completely consistent with those determined here.

### 3.2 Dependence of the log*K* ^H^ *_i_* on Ionic Strength and Temperature

The dependence of the protonation constants of *TXA* on ionic strength in NaCl, KCl, and (CH_3_)_4_NCl aqueous solutions was modeled by using two approaches: the extended Debye–Hückel type equation^7–10^ and the Specific ion Interaction Theory (SIT)^19–22^ according to eqs 9–13.

This allowed the calculation of the protonation constants at infinite dilution and the corresponding parameters of eqs 11, 12, and 13, for the modeling of their variation with respect to the supporting electrolyte concentration (i.e., ionic strength).

In the first approach (eqs 11 and 12 and Table 4), the data refer to the molar concentration scale (*I*/mol dm^–3^), while in the second (eq 13 and Table 5), the data refer to the molal one (*I*/mol kg^–1^).

**Table 4:** Protonation Constants at Infinite Dilution and Parameters for the Dependence on Ionic Strength of eq 12 and Temperature of eq 14 in the Molar Concentration Scale, at *p* = 0.1 MPa

equilibrium	log*K*_1_^H0^ ^a^	*c*_∞_ ^b^	*c*_0_ ^b^	*A*′^c^	*B*′^c^	*C*′^c^
H^+^ + *TXA* ^–^ = H(*TXA*)						
	10.566 ± 0.017^d^					
NaCl		0.231 ± 0.011^d^	0.691 ± 0.048	–0.0393 ± 0.0006	0.0004 ± 0.0003	0.00030 ± 0.00001
KCl		*C* = 0.41 ± 0.03^d^ ^,^ ^e^				
(CH_3_)_4_NCl		0.024 ± 0.031	0.701 ± 0.078			

equilibrium	log*K*_2_^H0^ ^f^	*c*_∞_	*c*_0_	*A*′	*B*′	*C*′
H^+^ + H*TXA* = H_2_(*TXA*)^+^						
	4.379 ± 0.007^d^					
NaCl		0.204 ± 0.004^d^	–0.076 ± 0.019	0.0006 ± 0.0004	0.0001 ± 0.0001	–0.0009 ± 0.0002
KCl		*C* = 0.105 ± 0.011^d^ ^,^ ^e^				
(CH_3_)_4_NCl		0.314 ± 0.011	–0.143 ± 0.026			

a log*K*_1_^H0^ = protonation constant at infinite dilution of the first protonation step, at the reference temperature (*T* = 298.15 K).

b *c*_∞_ and *c*_0_ are empirical parameters of the dependence on ionic strength of eq 12, for *I* → ∞ and *I* → 0, respectively.

c *A*′, *B*′ and *C*′ are empirical parameters of the dependence on temperature of eq 14, in the molar concentration scale.

d ±Std. Dev. on the whole set data containing the measurements in all experimental conditions (different ionic strength and temperature values; ∼50 measurements).

e Due to the short ionic strength interval investigated, the eq 11, with only the *C* parameter for the dependence on ionic strength, was used.

f log*K*_2_^H0^ = protonation constant at infinite dilution of the second protonation step, at the reference temperature (*T* = 298.15 K). Standard uncertainties (*u*): *u*(*T*) = 0.1 K and *u*(*p*) = 1 kPa.

**Table 5:** Protonation Constants at Infinite Dilution and Parameters for the Dependence on Ionic Strength of eq 13 and Temperature of eq 14 in the Molal Concentration Scale, at *p* = 0.1 MPa

equilibrium	log*K*_1_^H0^ ^a^	Δε_∞_ ^b^	Δε_0_ ^b^	Δ*H* ^0^ ^c^	Δε′^d^	Δ*C*_p_ ^e^	
H^+^ + *TXA* ^–^ = H(*TXA*)							
	10.566 ± 0.016^f^						
NaCl		0.199 ± 0.007^f^	0.759 ± 0.041	–49.9 ± 1.6	–3.4 ± 0.5	–0.29 ± 0.08	
KCl		Δε = 0.355 ± 0.05^f^ ^,^ ^g^					
(CH_3_)_4_NCl		–0.095 ± 0.024	0.530 ± 0.080				

equilibrium		log*K*_1_^H0^ ^h^	Δε_∞_	Δε_0_	Δ*H* ^0^	Δε′	Δ*C*_p_
H^+^ + H*TXA* = H_2_(*TXA*)^+^							
		4.378 ± 0.001^f^					
NaCl			0.167 ± 0.004^f^	–0.050 ± 0.020	1.0 ± 0.8	–1.7 ± 0.3	0.19 ± 0.04
KCl			Δε = 0.42 ± 0.03^f^ ^,^ ^g^				
(CH_3_)_4_NCl			0.023 ± 0.009	–0.200 ± 0.031			

a log*K*_1_^H0^ = protonation constant at infinite dilution of the first protonation step, at the reference temperature (*T* = 298.15 K).

b Δε_∞_ and Δε_0_ are the difference for the specific ion interaction parameters of the dependence on ionic strength of eq 13 (SIT approach).

c Δ*H* ^0^ is the protonation enthalpy at infinite dilution, in J K^–1^ mol^–1^ and in the molal concentration scale for the two protonation steps of *TXA*, eq 14.

d Δε′ is the parameter for the dependence on the ionic strength of Δ*H* ^0^.

e Δ*C*_p_ is expressed in J K^–1^ mol^–1^.

f ±Std. Dev. on the whole set data.

g Due to the short ionic strength interval investigated, the eq 13, with only Δε parameter for the dependence on ionic strength, was used.

h log*K*_2_^H0^ = protonation constant at infinite dilution of the second protonation step, at the reference temperature (*T* = 298.15 K). Standard uncertainties (*u*): *u*(*T*) = 0.1 K and *u*(*p*) = 1 kPa.

Concerning the dependence of the protonation constants on the temperature, from the data listed in Table 3, it is evident that log*K* ^H^ *_i_* values decrease by increasing the temperature and, for the log*K*_2_^H^ from *T* = 310.15 K, the values tend to decrease by increasing *T*.

The dependence of the log*K* ^H^ *_i_* values on *T*/K was modeled by also considering the term (*M*) of eq 9 and is expressed by eq 14. After the conversion in the molal concentration scale of both the ionic strengths and the protonation constants determined in NaCl_(aq)_ in the temperature range of 283.15 ≤ *T*/K ≤ 318.15, it was possible to calculate the protonation constants and the enthalpy change values at infinite dilution and at the reference temperature (*T* = 298.15 K).

To convert the ionic strength and protonation constants from the molar (*c*) to the molal (*m*) concentration scale, the expression *c*/*m* = *d*_0_ + *a*_1_ *c* + *a*_2_ *c* ^2^ proposed by De Stefano et al.^23^ was applied, where *d*_0_ is the solvent density at *T* = 273.15 K (reference temperature), with *d*_0_ = 0.99987 g cm^–3^ (for water). The *a*_1_ and *a*_2_ empirical parameters, calculated by Cardiano et al.^24^ for each supporting electrolyte using density literature data at different concentrations and temperatures, allow the conversion from one scale to the other. These data were already reported in a previous paper;^24^ for example, at *T* = 298.15 K, *a*_1_ = −0.017765 for NaCl and −0.107951 for (CH_3_)_4_NCl, while *a*_2_ = −6.525·10^–4^ for NaCl and 4.833·10^–4^ for (CH_3_)_4_NCl;^24^ for KCl, *a*_1_ is 0.026870 and a_2_ is −10.2578·10^–4^ (internal data).

From the data reported in Table 5, an exothermic process for the first protonation step and an endothermic one for the second were observed. These values are in perfect agreement with the literature finding,^25,26^ namely, ∼−50 kJ mol^–1^ for the primary amine group (log*K*_1_^H^)^27^ and ∼1 to 2 kJ mol^–1^ for the carboxylic one (log*K*_2_^H^).^28^

### 3.3 Medium Effect on the Protonation of TXA

The different behavior of the protonable groups of *TXA* with respect to variations in the supporting electrolyte concentration can be attributed to the different interaction properties of their ions. In particular, the chloride ion has the tendency to interact with the protonated amine group (−NH_3_^+^); instead, alkali metal ions tend to interact with oxygen donor ligands, forming weak ion pairs. Concerning tetraalkylammonium cations, the literature reports that they have very different natures with respect to alkali metal ions, since these, Na^+^ in particular, are structure-promoting ions (water molecules assume the structure of pure water), while tetraalkylammonium cations are structure-breaking ions, hence contributing to a different stabilization of the systems.^29^

The noninteracting nature of the tetraalkylammonium cations is linked to their hydrophobicity that in general tends to increase with the length of the alkyl chain.^29–32^ In our previous paper,^24^ where we investigated the acid–base properties and solubility of benzenopolycarboxylic ligands in NaCl aqueous solutions and tetraalkylammonium salts ((C_2_H_5_)_4_NI and (CH_3_)_4_NCl), we observed significantly different values for solubility and protonation constants in the three ionic media. Significant differences were also observed between the two tetraalkylammonium salts, with values in (C_2_H_5_)_4_NI_(aq)_ systematically higher with respect to those in NaCl_(aq)_ and (CH_3_)_4_NCl_(aq)_. These differences can be interpreted in terms of strength of interaction of Na^+^ and (CH_3_)_4_N^+^ with respect to (C_2_H_5_)_4_N^+^, which can be considered a noninteracting medium toward oxygen donor ligands.

The variation of the protonation constants with respect to the ionic strength (i.e., concentration of the supporting electrolyte) can be explained in terms of activity coefficient variation^33^ or of formation of weak complexes between the components of the supporting electrolyte and the ligand.^33^

Using this approach, from the protonation constants calculated at infinite dilution (i.e., extrapolated at zero ionic strength) and applying for *c*_0_ and *c*_1_ parameters (eq 11), the values valid in noninteracting medium (*c*_0_ = 0.11 and *c*_1_ = 0.20 ^11^) were calculated, and the difference of the log*K* ^H^ *_i_* values at the same supporting electrolyte concentration with respect to those determined in NaCl_(aq)_, KCl_(aq)_, and (CH_3_)_4_NCl_(aq)_ can be interpreted in terms of interaction of *TXA* with the components of the ionic media (i.e., Na^+^, K^+^, and (CH_3_)_4_N^+^).

This allowed, using the ES2WC^16^ computer program, the calculation of the formation constants of the weak complexes formed by these interactions. Moreover, using a similar approach with respect to the one used with eq 9 and adding a ternary term of both dependence on z* and *I*

16 $$D=d_{1}z^{*}I^{1/2}$$

where generally *d*_1_ = −0.075;^11^ it was also possible for the weak complexes to model the dependence on *I*/mol dm^–3^. Table 6 reports the formation constants of the weak complexes formed with Na^+^, K^+^, and (CH_3_)_4_N^+^. Similar calculations were also performed to check the possible formation of weak complexes with chloride, but these were systematically rejected by the ES2WC computer program, probably because of their very low stability.

**Table 6:** Formation Constants, at Infinite Dilution, and Parameters for the Dependence on Ionic Strength (eq 9) of the Weak Complexes of Tranexamic Acid with (CH_3_)_4_N^+^, Na^+^, and K^+^, at *p* = 0.1 MPa

equilibrium	log*K* ^0^ ^a^	*C* ^b^	*d*_1_ ^c^
(CH_3_)_4_N^+^ + *TXA* ^–^ = ((CH_3_)_4_N)(*TXA*)	–0.57 ± 0.06^d^	0.73 ± 0.01^d^	–0.10 ± 0.01^d^
Na^+^ + *TXA* ^–^ = Na(*TXA*)	–1.3 ± 0.1	0.68 ± 0.04	–0.10 ± 0.01
K^+^ + *TXA* ^–^ = K(*TXA*)	–1.1 ± 0.1	0.70 ± 0.03	–0.10 ± 0.01

a log*K* ^0^ = formation constant at infinite dilution of the complex species, at the reference temperature (*T* = 298.15 K).

b *C* is an empirical parameter of the dependence on ionic strength of the weak complex species; see eq 11.

c *d*_1_ is an empirical parameter of the dependence on ionic strength of the complex species; see eq 18.

d ±Std. Dev. on the whole set data. Standard uncertainties (*u*): *u*(*T*) = 0.1 K and *u*(*p*) = 1 kPa.

### 3.4 Complexing Ability of Tranexamic Acid toward M^2+^

The complexing ability of *TXA* toward selected bivalent metal cations (Ca^2+^, Mg^2+^, Zn^2+^) was investigated in NaCl_(aq)_ at different ionic strengths (0.15 ≤ *I*/mol dm^–3^ ≤ 1.00) and at *T* = 298.15 ± 0.15 K. For Sn^2+^, NaNO_3(aq)_ was used as a supporting electrolyte, since this tends to form stable complexes with chloride ions.^34^

The hydrolytic constants of the investigated metal cations are available in the literature.^34^ The pH range investigated by potentiometric titrations varied among the different systems, depending on the hydrolytic behavior of each metal ion and on the formation of insoluble species. For Ca^2+^ and Mg^2+^/*TXA* systems, the investigated pH range was 2.0–10.5, while for the Zn^2+^/*TXA*, it ranged from 2.0 to 8.0; over this pH value, precipitation occurred. By processing the potentiometric data by means of the BSTAC^16^ computer program, the M*TXA* complex species was obtained for each system, and in the case of the Zn^2+^/*TXA* system, the Zn*TXA*(OH) species was also determined.

By comparing the stability constant values of the Ca*TXA*, Mg*TXA*, and Zn*TXA* species, the following stability trend is observed: log*K*_ZnTXA_ (5.14) > log*K*_CaTXA_ (1.85) > log*K*_MgTXA_ (1.76). Table 7 reports the experimental stability constants at the different ionic strengths investigated.

**Table 7:** Experimental and Calculated Formation Constants^a^ in NaCl_(aq)_, at Different Ionic Strengths and *T* = 298.15 ± 0.15 K, Together with the Ionic Strength Dependence Parameters of the M^2+^/*TXA* Systems, *p* = 0.1 MPa

*I̅*/mol dm^–3^	logβ^a^	*I̅* ^b^/mol dm^–3^	logβ	*I̅*/mol dm^–3^	logβ	
	Ca(*TXA*)^+^		Mg(*TXA*)^+^		Zn(*TXA*)^+^	Zn(*TXA*)(OH)
0.147	1.85 ± 0.04^c^	0.146	1.76 ± 0.07^c^	0.144	5.14 ± 0.02^c^	–2.02 ± 0.01^c^
0.237	1.73 ± 0.04	0.236	1.91 ± 0.06	0.256	5.33 ± 0.02	–1.88 ± 0.02
0.475	1.70 ± 0.05	0.460	2.15 ± 0.05	0.513	5.82 ± 0.04	–1.49 ± 0.05
0.834	2.15 ± 0.08	0.855	2.28 ± 0.06	0.898	6.60 ± 0.09	–0.88 ± 0.09
0.95	1.96 ± 0.09	0.962	2.29 ± 0.07			

*I*/mol dm^–3^		logβ	logβ	*I*/mol dm^–3^	logβ	
		Ca(*TXA*)^+^	Mg(*TXA*)^+^		Zn(*TXA*)^+^	Zn(*TXA*)(OH)
	*C* ^d^	0.69 ± 0.16^e^	0.95 ± 0.08^e^		2.79 ± 0.12^e^	2.36 ± 0.12^e^
0		2.16 ± 0.08^e^	2.25 ± 0.07^e^	0	5.25 ± 0.03^e^	–1.84 ± 0.02^e^
0.15		1.77	1.89	0.15	5.15	–2.02
0.25		1.76	1.90	0.25	5.32	–1.89
0.50		1.81	2.02	0.50	5.80	–1.51
1.0		2.04	2.38	0.75	6.31	–1.11

a logβ = formation constants; refer to the equilibria (eqs 7 and 8).

b I̅ = average ionic strength.

c ±Std. Dev. on the whole set data containing the measurements in all experimental conditions (different ionic strengths; ∼25 measurements).

d *C* = empirical parameter of the dependence on ionic strength, eq 9.

e ±Std. Dev. on the whole set data. Standard uncertainties: *u*(*T*) = 0.1 K, *u*(*I*) = 0.001 mol dm^–3^, and *u*(*p*) = 1 kPa.

The analysis of the data confirms that Ca^2+^ and Mg^2+^ exhibit similar behaviors in terms of complexation toward *TXA*, as already observed for many other carboxylate systems.^25^

From Figure 4, it can be observed that the stability constant of the M*TXA* complex species increases with an increasing ionic strength.

**Figure 4:**
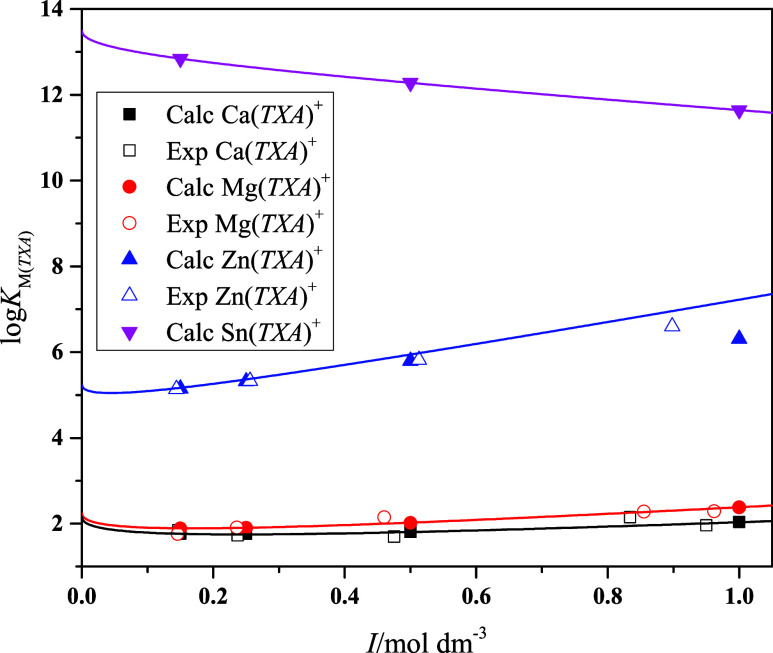
Trend of the log*K*_M(*TXA*)_ values of the different metal–ligand systems vs the ionic strength in NaCl_(aq)_ at *T* = 298.15 K, according to the ionic strength dependence parameters of eq 9, reported in Tables 7 and 8. Values represented by the filled symbols are calculated values, whereas those represented by open symbols are experimental. Data fitting, performed using eq 9, was carried out on experimental values.

The modeling of the formation constants as a function of ionic strength was carried out using a Debye–Hückel type equation (eq 9); Table 7 reports both the parameters describing the dependence on *I*/mol dm^–3^ and the calculated logβ values at different ionic strengths.

More clarifications must be done for the investigation of the Sn^2+^/*TXA* system. Initially, measurements were performed by means of potentiometric titrations, but independent of the experimental conditions (i.e., ionic strength and component concentrations), the formation of a precipitate occurred after the addition of some aliquots of titrant (NaOH: ∼0.1 mol dm^–3^). To overcome the limit of the insoluble species formation, the interactions between *TXA* ^–^ and Sn^2+^ were studied by means of differential pulse anodic stripping voltammetry (DP-ASV). This technique permitted the use of lower metal concentrations with respect to the ones employed for potentiometry (see Table 2 for further details), hindering the formation of the insoluble species up to pH ∼4.5.

The investigations were carried out over a metal concentration range of *c*_Sn_ 10^–7^–10^–5^ mol dm^–3^, while ligand concentrations were in the range of (3.0–5.0)·10^–3^ mol dm^–3^. This allowed us to collect experimental data in the ionic strength interval of 0.10–1.0 mol dm^–3^ in NaNO_3(aq)_ at *T* = 298.15 ± 0.15 K. The experimental data were elaborated by means of the LIANA computer program,^16^ employing eq 15, together with the mass balance equations for the metal cation (eq 16) and the ligand (eq 17). As for the potentiometric data, the dependence of the stability constants on the ionic strength was modeled using eq 9. The speciation model is featured by only the Sn­(*TXA*)^+^ species, whose logβ (at different ionic strengths and infinite dilution) values are listed in Table 8, highlighting a decreasing trend with increasing ionic strength.

**Table 8:** Formation Constant Values^a^ of the Sn­(*TXA*)^+^ Species Obtained by Voltammetry and Ionic Strength Dependence, by eq 9, in NaNO_3(aq)_, *T* = 298.15 K, and *p* = 0.1 MPa

species	logβ^0^ ^a^	z*^b^	*C* ^c^	*I* ^d^/mol dm^–3^		
				0.15	0.5	1.0
Sn(*TXA*)^+^ ^a^	13.5 ± 0.1^e^	4	–1.04 ± 0.20^e^	12.84	12.28	11.64

a log β^0^ = formation constants, refer to the equilibrium (eq 7).

b z* = ∑(charge)_reactants_^2^ – ∑(charge)_products_^2^.

c *C* = empirical parameter of the dependence on ionic strength, eq 9.

d *I* = ionic strength.

e ±Std. Dev. on the whole set data. Standard uncertainties: *u*(*T*) = 0.1 K, *u*(*I*) = 0.001 mol dm^–3^, and *u*(*p*) = 1 kPa.

Comparing the stability constants of the M­(*TXA*) species under the same conditions of ionic strength and temperature, the following stability trend occurs: log*K*_Sn*TXA*_ > log*K*_Zn*TXA*_ > log*K*_Mg*TXA*_ > log*K*_Ca*TXA*_.

The interpretation of metal–ligand interactions in this work is based on an electrostatic approach; nevertheless, the observed stability sequence can also be qualitatively rationalized by considering the electronic configurations and coordination tendencies of the different metal ions.

### 3.5 Sequestering Ability of Tranexamic Acid toward M^2+^

When binding equilibria are involved in different processes (environmental, biological, industrial, etc.) the choice of the “best” binding agent is crucial. Such an agent should be able to form stable complexes with the different metals and should be selective “for the target metal cation”.^35^ It is well-known that this capacity can be influenced by several factors such as pH, temperature, ionic strength, and the formation of “side species”, when the ligand of interest is dissolved in a multicomponent solution, containing other ligands and cations. In this case, the efficacy of the chelation is influenced by the competitive reactions, involving simultaneous equilibria in different conditions.^36^

In these conditions, both the selectivity and the sequestering ability of a chelant toward a cation cannot be easily estimated by the simple analysis of single sets of stability constants of metal/ligand complexes or by comparing the stability of a single species.

To overcome this problem, the use of the pL_0.5_ ^37^ parameter was proposed as a possible alternative to other already known methods of evaluation of the efficiency of the chelation of a ligand toward a cation.

The pL_0.5_, expressed by means of a dose–response type curve, can be represented by the equation

17 $$\chi_{M}=\frac{1}{1+10^{\left(\mathrm{pL}-{\mathrm{pL}}_{0.5}\right)}}$$

where the molar fraction of the complexed metal cation (present in trace 10^–12^ mol dm^–3^), is plotted vs pL, namely the −log *c*_L_ (*c*_L_ = analytical concentration of the ligand). It is important to highlight that the pL parameter considers all the competitive reactions involving all the components present in the system (e.g., hydrolysis, protonation, formation of other complexes), but they were excluded from the calculation of the pL_0.5_, namely, the ligand concentration necessary to sequester the 50% of the metal concentration.

The pL_0.5_ values calculated for Ca^2+^ and Mg^2+^/*TXA* systems showed a scarcely sequestering ability of *TXA* toward these two metal cations, while it is relevant for Zn^2+^ and Sn^2+^, in particular; the significant data obtained for the last two cations are listed in Table 9. It is evident that the sequestering ability of *TXA* vs Sn^2+^ decreases with increasing ionic strength and increases with increasing pH values until pH ∼4.0, after which the pL_0.5_ values decrease. In the case of the sequestration of Zn^2+^, only at pH 7.4, a significant capacity was highlighted, and this increases with increasing ionic strength value.

**Table 9:** pL_0.5_ Values Calculated by Means of eq 19 for the Sn^2+^ and Zn^2+^/*TXA* Systems at Different pH and Ionic Strength Values, at *T* = 298.15 K and *p* = 0.1 MPa^a^

metal	*I*/mol dm^–3^	pH	pL_0.5_	metal	*I*/mol dm^–3^	pH	pL_0.5_
Sn^2+^				Zn^2+^			
	0.15	3.0	3.86		0.15	7.4	2.53
		4.0	4.35		0.5	7.4	3.00
		7.4	1.44		1.0	7.4	3.36
	0.5	3.0	3.25				
		4.0	3.85				
		7.4	1.01				
	1.0	3.0	2.43				
		4.0	3.16				
		7.4	0.39				

a *I* = ionic strength; pL_0.5_ = the ligand concentration necessary to sequester the 50% of the metal concentration. Standard uncertainties: *u*(*T*) = 0.1 K, *u*(*I*) = 0.001 mol dm^–3^, and *u*(*p*) = 1 kPa.

### 3.6 Simulation in Blood Plasma

A thorough understanding of thermodynamic formation parameters is essential for addressing issues in several application fields, including industrial, biological (e.g., metal detoxification processes or drug delivery), and environmental contexts (such as the remediation of contaminated sites). Furthermore, it enables the identification of not only the chemical species in which a given component is distributed within a real system (biological or natural) but also their abundance at specific component concentrations.

Speciation studies also allow the evaluation of the influence of secondary components on the formation and distribution of species within the system under investigation. In light of these considerations, a simulation was carried out to better understand the distribution of species of the different systems (Ca^2+^/*TXA*, Mg^2+^/*TXA*, and Zn^2+^/*TXA*) under conditions reproducing human blood plasma composition.

Regarding plasma simulations, several models have been reported in the literature considering different organic and inorganic components. In the present study, the model proposed by Marques et al.^38^ was adopted. This model includes various inorganic salts (sodium chloride, sodium bicarbonate, potassium chloride, dipotassium hydrogen phosphate trihydrate, magnesium chloride hexahydrate, calcium chloride dihydrate, and sodium sulfate), while the organic fraction is simulated by tris­(hydroxymethyl)­aminomethane (THAM).

In particular, all possible binary and ternary interactions among plasma components were considered, including the corresponding formation constants (for both ligand protonation and complex formation). The hydrolysis of metal cations (M^n+^ = Na^+^, K^+^, Ca^2+^, and Mg^2+^) was neglected, as these reactions become significant at pH values higher than 10. The input models used to simulate the behavior of the Ca^2+^/*TXA*, Mg^2+^/*TXA*, and Zn^2+^/*TXA* systems also account for the main organic and inorganic components naturally present in plasma and their average concentrations. Moreover, the formation constants of all of the secondary species potentially generated by these components were included.

Figure 5a,b shows two different graphical representations of the simulations performed in plasma at pH = 7.4. Figure 5a presents a pie chart obtained by considering the sum of the species of each system (Ca^2+^/*TXA*, Mg^2+^/*TXA*, and Zn^2+^/*TXA*), which are compared with one another. Figure 5b illustrates the distribution of the species involving only the Zn^2+^/*TXA* system.

**Figure 5:**
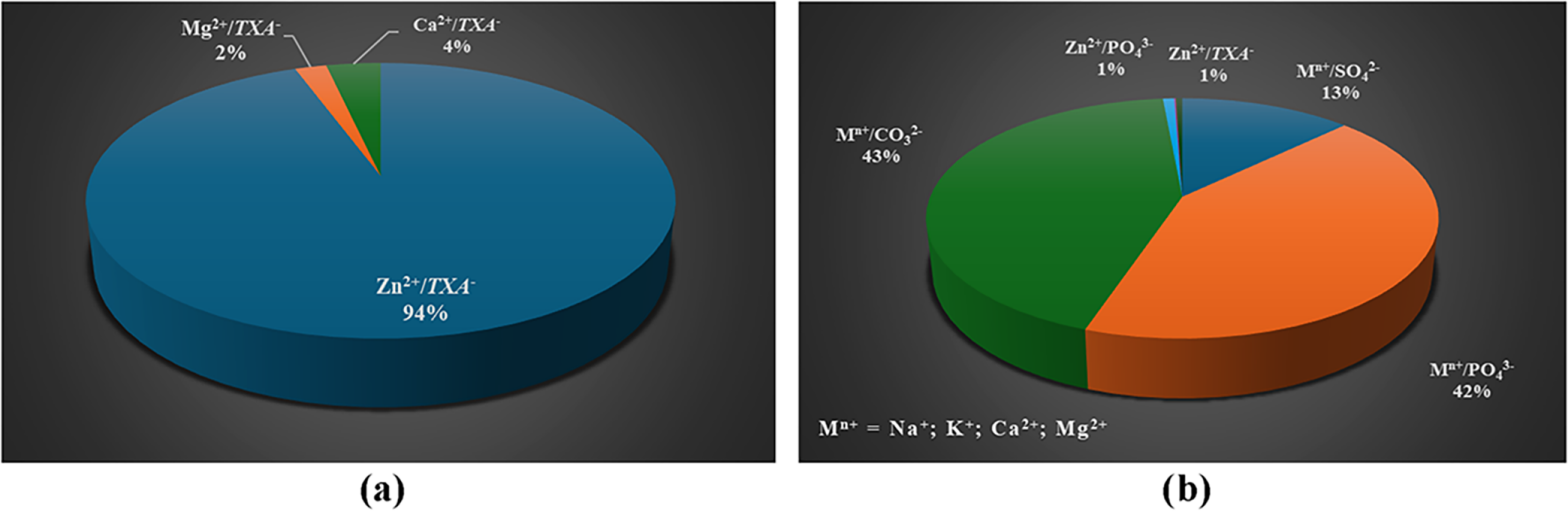
Pie charts of the percentage of *TXA* species formation in human plasma: (a) formation percentages of the Ca^2+^/*TXA* ^–^, Mg^2+^/*TXA* ^–^, and Zn^2+^/*TXA* ^–^ species and (b) sum of the Zn^2+^/*TXA* ^–^ species and those formed by the secondary components in human plasma^38^ at pH = 7.4.

Analysis of the latter system reveals a clear predominance of M/CO_3_^2–^ and M/PO_4_^3–^ complexes, accounting for approximately 43 and 42% of the total distribution, respectively. The formation of complexes with other inorganic anions is more limited (approximately 13% overall). Complexes formed between *TXA* and Zn^2+^ account for ∼1% of the total species.

It should be emphasized that these considerations are valid only under the simulated conditions since variations in component concentrations lead to changes in species distribution and formation percentages. Nevertheless, these simulations represent a useful tool for highlighting the relative abundances of the different species under physiological conditions.

Concentrations of the components: *c*_Na_ = 115.9·10^–3^ mol dm^–3^; *c*_K_ = 4.2·10^–3^ mol dm^–3^; *c*_Ca_ = 1.6·10^–3^ mol dm^–3^; *c*_Mg_ = 1.2·10^–3^ mol dm^–3^; *c*_Cl^–^_ = 170.2·10^–3^ mol dm^–3^; *c*_SO4_ = 0.4·10^–3^ mol dm^–3^; *c*_PO_4__ = 0.9·10^–3^ mol dm^–3^; *c*_CO3_ = 3.4·10^–3^ mol dm^–3^; *c*_THAM_ = 31.4·10^–3^ mol dm^–3^; *c*_Zn_ = 0.02·10^–3^ mol dm^–3^; and *c*_*TXA*_ = 2.0·10^–3^ mol dm^–3^.

It should be emphasized that the present simulations were performed using a simplified synthetic plasma model and laboratory-scale *TXA* concentrations; therefore, they do not fully reproduce physiological conditions; consequently, the effects of competitive endogenous ligands (such as serum albumin) and the variability of plasma levels depending on the route of administration are not explicitly included.

## 4 Conclusions

This paper presents studies providing insights into the thermodynamic behavior of tranexamic acid, particularly its protonation properties and its interactions with different metal cations: Ca^2+^, Mg^2+^, and Zn^2+^ in NaCl_(aq)_ and Sn^2+^ in NaNO_3(aq)_. Knowledge of the interactions between the metals and the ligand is essential for delineating speciation models and can provide useful information on the potential use of *TXA* as a sequestering agent to remove or release the metal cations from natural and biological fluids. The main findings of this study can be summarized as follows:

- The acid–base properties of tranexamic acid were defined in different ionic media, NaCl_(aq)_, KCl_(aq)_, and (CH_3_)_4_NCl_(aq)_, over an ionic strength range of 0.10 ≤ *I*/mol dm^–3^ ≤ 4.5. The temperature was fixed at *T* = 298.15 ± 0.15 K in KCl_(aq)_ and (CH_3_)_4_NCl_(aq)_, while it was varied in the range of 283.15 ≤ *T*/K ≤ 318.15 in NaCl_(aq)_.
- The formation constants of the weak Na*TXA*, K*TXA*, and (CH_3_)_4_N*TXA* complex species of the ligand with the cations of the supporting electrolyte were determined.
- Potentiometric titrations revealed speciation models very similar for all the M^2+^/*TXA* systems, with the formation of M*TXA* species, and in the case of the Zn^2+^/*TXA* ^–^ system, also the Zn*TXA*(OH) species.
- Because of the formation of the insoluble species at acidic pH values, the Sn^2+^/*TXA* system was studied by voltammetry, and the Sn*TXA* species was determined in NaNO_3(aq)_.
- The dependence of protonation and of stability constants on ionic strength was modeled by two approaches: the Debye–Hückel type equation and the Specific ion Interaction Theory (SIT).
- Tranexamic acid shows fairly good sequestering ability toward Zn^2+^ and Sn^2+^, and this changes a lot with the ionic strength and pH values. For example, the pL_0.5_ values, at *T* = 298.15 K and pH = 7.4, vary from 2.53 to 3.36, at *I* = 0.15 and 1.00 mol dm^–3^, respectively, for zinc; they vary in the same conditions from 1.44 to 0.39 for tin­(II).
